# Early antituberculosis drug exposure in hospitalized patients with human immunodeficiency virus‐associated tuberculosis

**DOI:** 10.1111/bcp.14207

**Published:** 2020-02-17

**Authors:** Charlotte Schutz, Maxwell Chirehwa, David Barr, Amy Ward, Saskia Janssen, Rosie Burton, Robert J. Wilkinson, Muki Shey, Lubbe Wiesner, Paolo Denti, Helen McIlleron, Gary Maartens, Graeme Meintjes

**Affiliations:** ^1^ Wellcome Centre for Infectious Diseases Research in Africa Institute of Infectious Disease and Molecular Medicine, University of Cape Town Observatory South Africa; ^2^ Department of Medicine University of Cape Town Observatory South Africa; ^3^ Division of Clinical Pharmacology, Department of Medicine University of Cape Town Cape Town South Africa; ^4^ Wellcome Trust Liverpool Glasgow Centre for Global Health Research University of Liverpool Liverpool UK; ^5^ Amsterdam University Medical Centre University of Amsterdam Amsterdam Netherlands; ^6^ Khayelitsha Hospital, Department of Medicine Cape Town South Africa; ^7^ Department of Infectious Diseases Imperial College London UK; ^8^ The Francis Crick Institute London UK

**Keywords:** human immunodeficiency virus, tuberculosis, treatment, pharmacokinetics

## Abstract

**Aims:**

Patients hospitalized at the time of human immunodeficiency virus‐associated tuberculosis (HIV‐TB) diagnosis have high early mortality. We hypothesized that compared to outpatients, there would be lower anti‐TB drug exposure in hospitalized HIV‐TB patients, and amongst hospitalized patients exposure would be lower in patients who die or have high lactate (a sepsis marker).

**Methods:**

We performed pharmacokinetic sampling in hospitalized HIV‐TB patients and outpatients. Plasma rifampicin, isoniazid and pyrazinamide concentrations were measured in samples collected predose and at 1, 2.5, 4, 6 and 8 hours on the third day of standard anti‐TB therapy. Twelve‐week mortality was ascertained for inpatients. Noncompartmental pharmacokinetic analysis was performed.

**Results:**

Pharmacokinetic data were collected in 59 hospitalized HIV‐TB patients and 48 outpatients. Inpatient 12‐week mortality was 11/59 (19%). Rifampicin, isoniazid and pyrazinamide exposure was similar between hospitalized and outpatients (maximum concentration [C_max_]: 7.4 *vs* 8.3 μg mL^–1^, *P =* .223; 3.6 *vs* 3.5 μg mL^–1^, *P =* .569; 50.1 *vs* 46.8 μg mL^–1^, *P =* .081; area under the concentration–time curve from 0 to 8 hours: 41.0 *vs* 43.8 mg h L^–1^, *P* = 0.290; 13.5 *vs* 12.4 mg h L^–1^, *P =* .630; 316.5 *vs* 292.2 mg h L^–1^, *P =* .164, respectively) and not lower in inpatients who died. Rifampicin and isoniazid C_max_ were below recommended ranges in 61% and 39% of inpatients and 44% and 35% of outpatients. Rifampicin exposure was higher in patients with lactate >2.2 mmol L^–1^.

**Conclusion:**

Mortality in hospitalized HIV‐TB patients was high. Early anti‐TB drug exposure was similar to outpatients and not lower in inpatients who died. Rifampicin and isoniazid C_max_ were suboptimal in 61% and 39% of inpatients and rifampicin exposure was higher in patients with high lactate. Treatment strategies need to be optimized to improve survival.

**What is already known about this subject**

- Patients hospitalized with human immunodeficiency virus‐associated tuberculosis (HIV‐TB) have high mortality despite treatment and often present with a clinical picture compatible with sepsis.
- Deaths occur early and there is paucity of data regarding antitubercular drug exposure in hospitalized critically ill HIV‐TB patients.

**What this study adds**

- Rifampicin, isoniazid and pyrazinamide exposure in hospitalized HIV‐TB patients and outpatients on day 3 of standard treatment are described.
- Hospitalized HIV‐TB patients do not have lower exposure than outpatients; however, many have suboptimal concentrations, which could play a role in mortality.
- This could inform treatment strategies in hospitalized HIV‐TB patients.

## 1 INTRODUCTION

Tuberculosis (TB) is the leading cause of hospitalization and in‐hospital death in human immunodeficiency virus (HIV)‐infected people worldwide.^1^, ^2^ In high‐burden settings hospitalized patients with HIV‐associated TB (HIV‐TB) have case fatality rates between 11 and 32%.^3^, ^4^, ^5^, ^6^, ^7^, ^8^ The majority of these deaths occur within 2 weeks^3^, ^4^, ^5^, ^8^ and in postmortem series inpatient HIV‐TB deaths are reported at a median of 4–5 days after admission^9^, ^10^, with 50% of deaths occurring in patients already on anti‐TB therapy.^11^

Severe HIV‐TB may present with clinical features of bacterial sepsis.^12^, ^13^, ^14^ In high‐burden settings *Mycobacterium tuberculosis* bloodstream infection is the most common diagnosis in HIV‐infected patients presenting to hospital with a clinical syndrome of sepsis.^15^, ^16^, ^17^, ^18^ Analogous to sepsis, there are many factors in severe HIV‐TB that could reduce drug exposure, such as impaired absorption of orally administered drugs due to delayed gastric emptying and decreased perfusion of the gastrointestinal tract, increased volume of distribution due to fluid shifts, and augmented renal clearance.^19^, ^20^ Other factors in advanced HIV infection such as intestinal TB, HIV‐related enteropathy, and gastrointestinal opportunistic infections and macro‐ or micronutrient deficiencies^21^, ^22^, ^23^ could contribute to reduced drug exposure. Limited existing data suggest that anti‐TB drug exposure in critically ill patients is inadequate.^24^ Elevated blood lactate is used as a marker of sepsis severity^25^ and is associated with mortality in hospitalized patients with HIV‐TB.^5^

HIV infection has a variable effect on anti‐TB drug concentrations across studies, with some studies showing lower concentrations than in HIV‐negative patients.^26^, ^27^, ^28^ There are few pharmacokinetic studies in HIV‐TB that assess relationships between drug exposure and clinical outcomes.^29^, ^30^, ^31^

Rifampicin is a potent inducer of drug metabolizing liver enzymes^32^ and also undergoes auto‐induction.^33^ The majority of rifampicin pharmacokinetic studies have been performed after administration of multiple doses when autoinduction is advanced,^27^ yet mortality in hospitalized HIV‐TB patients occurs early. In the parent cohort of this pharmacokinetic (PK) study 37% of deaths occurred within 7 days of enrolment.^34^ Preliminary evidence suggest that higher‐dose than the currently recommended 10 mg kg^–1^ daily may improve survival in HIV‐TB patients with low CD4 counts.^35^

We performed intensive PK studies on the third day of anti‐TB therapy, administered at standard doses, in hospitalized patients with HIV‐TB and outpatient controls and determined 12‐week mortality in hospitalized patients. We compared exposure of rifampicin, isoniazid and pyrazinamide between inpatients and outpatients, between inpatients who survived and those who died within 12 weeks, and between inpatients presenting with an elevated lactate (a marker of sepsis severity) and those presenting with normal lactate. We hypothesized that exposure to rifampicin, isoniazid and pyrazinamide would be lower in inpatients than outpatients; lower in inpatients who died within 12 weeks compared to survivors, and lower in inpatients presenting with elevated venous lactate compared to those presenting with normal lactate.

## 2 METHODS

### 2.1 Study design and study population

We enrolled hospitalized HIV‐infected adults with a CD4 count of ≤350 cells μL^–1^ starting treatment for active TB at Khayelitsha Hospital and ambulant outpatients (HIV‐infected and uninfected) at Ubuntu clinic, Site B Khayelitsha, Cape Town, South Africa between November 2014 and November 2016. Inpatients were recruited as part of an observational cohort study investigating causes of mortality in hospitalized patients with HIV‐TB. HIV‐infected adults aged 18 years or older, with a suspected new diagnosis of TB were enrolled at presentation to hospital and PK studies were performed in a subgroup within the routine hospital service on the third day of anti‐TB therapy. Patients who survived to the third day of TB treatment were enrolled sequentially for PK studies, provided they still required inpatient care, did not require transfer to a tertiary care facility for intensive care or investigations and there were adequate staff to fulfil the parent study's operational requirements and perform PK study. Outpatients were enrolled at treatment initiation and returned for PK studies on the third day of therapy. Patients were enrolled regardless of antiretroviral therapy status or type. Outpatients were HIV‐infected or HIV‐uninfected. Clinical data and baseline blood tests were obtained at enrolment. Twelve‐week vital status was ascertained for inpatients.

#### 2.1.1 Anti‐TB therapy and PK study methods

Standard combination anti‐TB therapy for drug sensitive TB was administered according to weight as per the South African Department of Health National Tuberculosis Management guidelines^36^ and consisted of 4‐drug fixed‐dose combination (FDC) tablets containing rifampicin (150 mg), isoniazid (75 mg), pyrazinamide (400 mg) and ethambutol (275 mg). In the first 8 weeks of treatment, patients weighing 30–37 kg received 2 FDC tablets per dose, while those weighing 38–54, 55–70 or >70 kg received 3, 4 or 5 tablets respectively. One inpatient had crushed tablets (mixed with water) inserted via a nasogastric tube. Two inpatients with renal impairment received separate rifampicin, isoniazid, pyrazinamide and ethambutol tablets to allow alternate day dosing of ethambutol. Patients received the FDC formulation in use at the hospital and clinic at the time the study was conducted. All outpatient controls and 31/59 (52.5%) of hospitalized patients received Rifafour e‐275 (Sanofi) and the remaining hospitalized patients received RITIB (Pharmacare Limited).

Participants were fasted overnight and were offered a standardized breakfast after the 1‐hour sample and a standardized lunch between the 4‐ and 6‐hour samples. The study team administered the third dose of anti‐TB therapy and collected samples immediately before (0 h) and at 1, 2.5, 4, 6 and 8 hours after the dose. Timing of samples were calculated from the time the dose was administered and all samples were collected within a 10‐minute window (±5 min). A cold chain was maintained by placing blood samples in crushed ice immediately after collection, spinning in a cold centrifuge (8°C) and flash freezing plasma aliquots in dry ice within 30 minutes of collection. Plasma aliquots were transported and stored in a –80°C freezer at the end of each day.

Rifampicin, isoniazid and pyrazinamide concentrations were measured on stored plasma using high‐performance liquid chromatography coupled to tandem mass spectrometry at the Division of Clinical Pharmacology Laboratory, University of Cape Town. The combined accuracy and precision statistics of the low‐, medium‐, and high‐quality control samples during analysis (*n* = 22) of the rifampicin assay were between 99.7% and 100.8%, and 4.7% and 7.7%, respectively. The combined accuracy and precision statistics of the low‐, medium‐ and high‐quality control samples during analysis (*n* = 22) of the isoniazid assay were between 98.3% and 100.4%, and 3.0% and 5.1%, respectively. The combined accuracy and precision statistics of the low‐, medium‐ and high‐quality control samples during analysis (*n* = 22) of the pyrazinamide assay were between 88.1% and 92.3%, and 2.9% and 3.6%, respectively. Baseline blood tests including venous lactate measurements were performed at the National Health Laboratory Services.

### 2.2 Ethical approval

The study was approved by the University of Cape Town Human Research Ethics Committee (UCT HREC reference: 057/2013) and written informed consent was obtained for the PK substudy. Eligible inpatients with a decreased level of consciousness were enrolled and followed up daily until they regained capacity to participate in the informed consent process. Permission was sought from the UCT HREC to use information of participants who died prior to providing informed consent.

### 2.3 Statistical analysis

Noncompartmental analysis was performed using Stata/SE 13.1 for Mac (StataCorp, College Station, TX, USA) and all other comparative statistics were performed and plots created using R version 3.4.4 and the R Studio interface version 1.0.143.^37^, ^38^ Maximum concentration (C_max_) is defined as the maximum plasma concentration reached after administration of the third dose of anti‐TB therapy and within 8 hours. Comparisons of C_max_ and area under the concentration–time curve from 0 to 8 hours (AUC_0–8_) were made between inpatients and outpatients, between inpatients who survived and those who died, and between inpatients presenting with elevated lactate concentrations and those presenting with normal lactate. We compared groups using the Wilcoxon rank sum, Kruskal–Wallis, Pearson's χ^2^ or Fisher's exact test, where appropriate, and report median values with interquartile range (IQR) or number and percentage. We compared HIV‐positive outpatient controls to HIV‐negative outpatient controls. There were no differences in PK parameters of HIV‐positive *vs* HIV‐negative outpatients (Table S5) and this group was not disaggregated for any of the other analyses. Lactate was also compared to PK variables as a continuous variable. Correlations were performed on log or square root transformed variables using Pearson's correlation test or Spearman's rank correlation where appropriate. In hospitalized patients we calculated the odds ratio for survival per doubling of lactate concentration using a logistic regression model and log2 transformed lactate concentration. We did not adjust for other clinical variables. We performed correlation tests (Pearson or Spearman's correlation) to assess relationships between PK variables, creatinine clearance and conjugated bilirubin, and pyrazinamide exposure with 2 inflammatory markers (C‐reactive protein and procalcitonin). Concentrations below the lower limit of quantification (LLQ) were imputed at half the value of the LLQ. Missing concentrations were imputed using the slope of the relevant drug's log concentration curve for the patient when possible (Tables S1 and S2). The LLQ for rifampicin, isoniazid and pyrazinamide was 0.117, 0.105 and 0.203 μg mL^–1^, respectively.

Drug concentrations were log‐transformed and the geometric mean was calculated by exponentiating the mean of the log‐transformed values. We used published reference ranges of drug concentrations that can be expected after administration of standard doses of anti‐TB therapy for comparison for comparison of our C_max_ results (8–24 μg mL^–1^ for rifampicin, 3–6 μg mL^–1^ for isoniazid and 20–60 μg mL^–1^ for pyrazinamide).^31^, ^32^, ^33^

## 3 RESULTS

### 3.1 Outcomes of the parent study and baseline characteristics

The parent study enrolled 576 hospitalized patients with HIV‐TB and the 12‐week mortality was 124/576 (22%) at a median of 12.5 days from enrolment.^34^

**Figure 1:**
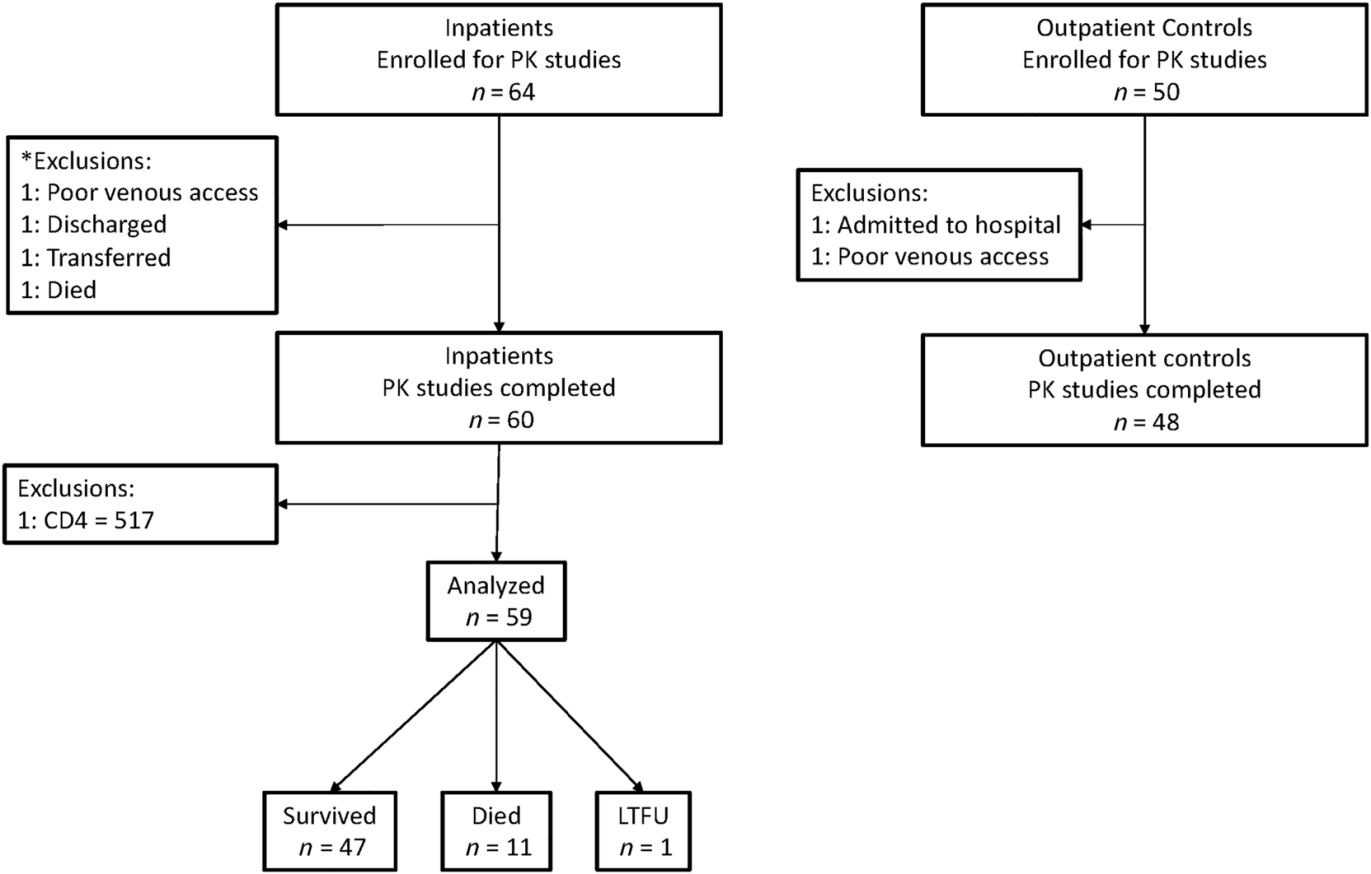
Study flow chart: hospitalized human immunodeficiency virus (HIV)‐infected adults with a CD4 count of ≤350 cells μL^–1^ starting tuberculosis treatment in hospital and ambulant outpatients (HIV‐infected and uninfected) were enrolled for intensive pharmacokinetic (PK) studies. Inpatients were enrolled at presentation and PK studies were performed within the routine hospital service on the third day of antituberculosis therapy, provided they still required inpatient care and did not need transfer for intensive care. Outpatients were enrolled at treatment initiation and returned for PK studies on the third day of therapy. Twelve‐week mortality was ascertained for inpatients. *Exclusions are only listed if participants had consented to take part in the study and PK study could not be performed. We did not document all patients who qualified to take part in the PK study and could not be included due to logistical reasons such as early deaths, transfers to tertiary facilities and staff availability

Intensive PK studies were performed in a subgroup of 60 inpatients and in 48 outpatients with TB. One inpatient was excluded due to a high CD4 count and an alternative diagnosis of mycetoma. We analysed data from 59 inpatients and 48 outpatients (Figure 1). Outpatients included 19/48 (40%) HIV‐uninfected patients. The median CD4 counts for inpatients and HIV‐infected outpatients were 58 and 146 cells μL^–1^, respectively (Table 1). Twelve‐week mortality for inpatients was 11/59 (19%) with median days from PK study to death = 40 days (interquartile range = 8–60 days). One inpatient was lost to follow up after 2 months.

**Table 1:** Baseline characteristics of outpatient controls with TB and hospitalized patients with HIV‐TB who had intensive pharmacokinetic studies performed on the third day of anti‐TB therapy

	Outpatient controls	Hospitalized	*P*
	*n =* 48	*n =* 59	
First episode of TB	32 (71.1)	35 (59.3)	.286
Sex, male	36 (75.0)	28 (47.5)	**.005**
Age, years	36 [32, 42]	37 [32, 41]	.980
HIV infected	29 (60.4)	59 (100)	‐
aHIV viral load, log copies mL^–1^	5.4 [4.3, 5.5]	5.0 [3.3, 5.7]	.684
bCD4 count, cells μL^–1^	146 [37, 233]	53 [16, 129]	**.007**
cCurrent antiretroviral therapy	5 (17.2)	20 (34.5)	.169
dMTB on TB blood culture	‐	16 (28.1)	‐
Glasgow coma score <15 at presentation	0 (0.0)	12 (20.3)	**.003**
eHeight, m	1.69 [1.64, 1.75]	1.64 [1.59, 1.70]	**.002**
Weight, kg	57.2 [52.0, 62.3]	54.5 [48.0, 60.8]	**.039**
fBody mass index, kg m^–2^	19.6 [18.3, 21.8]	19.3 [17.6, 22.4]	.561
Body mass index <18.5 kg m^–2^	15 (31.2)	21 (41.2)	.681
Body mass index 18.5 – 24.9 kg m^–2^	29 (60.4)	26 (51.0)	
Body mass index >25 kg m^–2^	4 (8.3)	4 (6.8)	
Random glucose, mmol L^–1^	4.5 [4.1, 5.2]	5.2 [4.7, 5.9]	**.002**
Lactate, mmol L^–1^	‐	1.6 [1.1, 2.3]	‐
C‐reactive protein, mg L^–1^	78.5 [46.8, 134.5]	192.0 [105.1, 264.5]	**<.001**
Procalcitonin, μg mL^–1^	‐	3.7 [0.6, 17.2]	‐
Aspartate amino transferase, U L^–1^	29.0 [21.0, 49.8]	50.0 [34.0, 82.3]	**<.001**
Alanine amino transferase, U L^–1^	19.5 [13.0, 29.0]	27.0 [18.0, 47.0]	**.012**
Gamma‐glutamyl transferase, U L^–1^	49.5 [35.8, 93.3]	77.0 [46.5, 144.5]	**.005**
Alkaline phosphatase, U L^–1^	88.0 [74.0, 117.5]	106.0 [75.5, 154.3]	**.043**
Total bilirubin, μmol L^–1^	10.0 [6.8, 14.0]	10.0 [6.0, 14.5]	.967
Conjugated bilirubin, μmol L^–1^	6.0 [4.0, 8.0]	6.0 [3.0, 9.0]	.607
Total protein, g L^–1^	81.0 [75.0, 87.5]	80.0 [69.5, 85.0]	.068
Albumin, g L^–1^	33.5 [32.0, 37.0]	25.0 [21.0, 29.5]	**<.001**
Creatinine, μmol L^–1^	62.0 [51.50, 72.5]	95.0 [63.0, 142.0]	**<.001**
Creatinine clearance, mL minute^–1^	120.5 [96.9, 145.7]	68.2 [45.2, 96.1]	**<.001**
Haemoglobin, g dL^–1^	11.1 [9.6, 12.0]	8.7 [7.1, 9.9]	**<.001**
White cell count, ×10^9^ L^–1^	7.1 [5.7, 9.6]	7.2 [5.1, 9.8]	.770
Platelets, ×10^9^ L^–1^	424.0 [316.0, 505.0]	291.0 [199.0, 355.0]	**<.001**
Absolute neutrophil count, ×10^9^ L^–1^	4.80 [3.53, 6.91]	5.43 [3.58, 8.41]	.241
Absolute lymphocyte count, ×10^9^ L^–1^	1.36 [1.04, 1.82]	0.66 [0.36, 0.95]	**<.001**
Absolute monocyte count, ×10^9^L^–1^	0.69 [0.50, 0.83]	0.34 [0.18, 0.62]	**<.001**
Rifampicin dose, mg kg^–1^	10.3 [9.2‐10.9]	10.0 [9.2, 11.1]	.607
Isoniazid dose, mg kg^–1^	5.2 [4.6‐5.5]	5.0 [4.6, 5.6]	.754
Pyrazinamide dose, mg kg^–1^	27.6 [24.4‐29.1]	26.7 [24.4, 29.6]	.525

Continuous variables are presented as median with [interquartile range] and categorical variables as *n* (%).

*P*‐value represents result of the nonparametric test comparison (Wilcoxon rank sum test for continuous variables and Fisher's exact or Pearson's χ^2^ test for categorical variables).

TB: tuberculosis; HIV: human immunodeficiency virus; CD4: cluster of differentiation 4; MTB: *Mycobacterium tuberculosis*

a HIV viral load for hospitalized patients (*n =* 59) and HIV‐infected outpatients (*n =* 29)

b CD4 count for all hospitalized patients (*n =* 59) and HIV‐infected outpatients (*n =* 29)

c Current antiretroviral therapy indicated as a proportion of HIV‐infected outpatients (*n =* 29).

d Mycobacterial blood culture was not performed in outpatients.

e Height was missing for 8 hospitalized patients: 4 survivors, 3 patients who died, 1 lost to follow up.

f Body mass index was not calculated for patients with missing height.

On baseline blood tests there were significant differences between inpatients and outpatient controls, including significantly lower CD4 count, haemoglobin, creatinine clearance and albumin, and significantly higher liver enzymes and C‐reactive protein in inpatients (Table 1). Inpatients and outpatients received similar doses (mg kg^–1^) of rifampicin, isoniazid and pyrazinamide and there was a similar distribution of patients in different weight categories (Table 1). There were fewer differences between hospitalized patients who died and those who survived 12 weeks of follow up (Table S3).

### 3.2 C_max_

**Figure 2:**
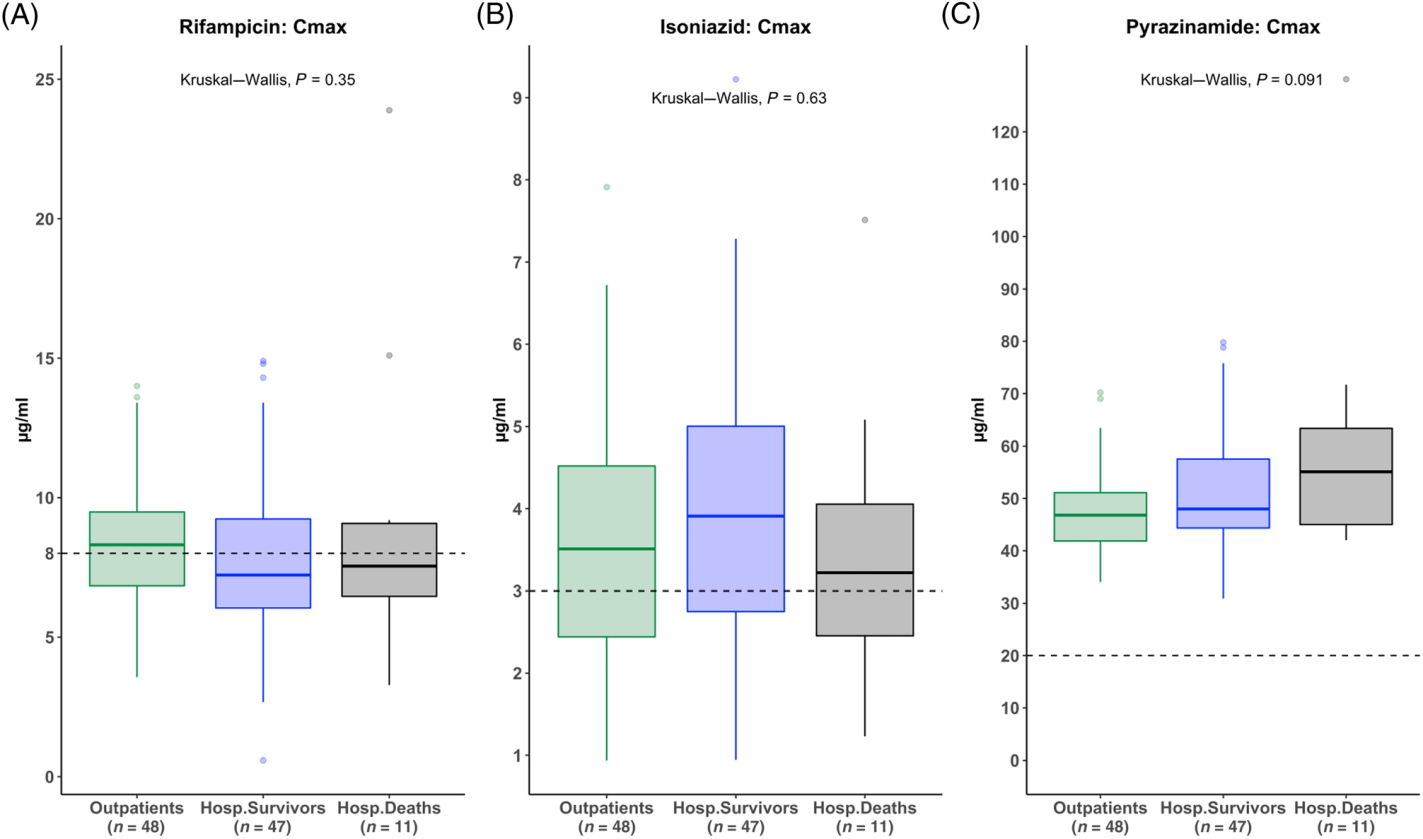
Rifampicin, isoniazid and pyrazinamide peak concentrations (C_max_) on the third day of antituberculosis therapy: boxplots of rifampicin, isoniazid and pyrazinamide maximum concentrations are presented in μg mL^–1^ for outpatients (green), hospitalized patients who survived 12‐week follow up (blue) and hospitalized patients who died within 12 weeks (black). *P* value: Kruskal–Wallis test comparing C_max_ values across 3 groups. Dashed horizontal lines represent the minimum threshold of the reference range: 8 μg mL^–1^ for rifampicin, 3 μg mL^–1^ for isoniazid and 20 μg mL^–1^ for pyrazinamide. C_max_: maximum (peak) concentration; Outpatients: ambulant tuberculosis patients attending outpatient clinic for treatment; Hosp.Survivors: hospitalized survivors; Hosp.Deaths: hospitalized patients who died within 12 weeks of enrolment

Comparing hospitalized patients to outpatients, neither the median rifampicin C_max_ (7.4 *vs* 8.3 μg mL^–1^, *P =* .223), nor the median isoniazid C_max_ (3.6 *vs* 3.5 μg mL^–1^, *P =* .569) were significantly different. The median pyrazinamide C_max_ in hospitalized patients was higher than outpatients (50.1 *vs* 46.8 μg mL^–1^, *P =* .081) but this did not reach statistical significance (Table 2 and Figure 2). Rifampicin C_max_ was below the minimum threshold of the reference range of 8 μg mL^–1^ in 36/59 (62%) and 21/48 (44%), of hospitalized patients and outpatients (95% confidence interval of the difference in proportions [95% CI]: –3.4, 37.9; *P =* .079), respectively. Isoniazid C_max_ was below the minimum recommended 3 μg mL^–1^ in 23/59 (39%) and 17/48 (35%) of hospitalized and outpatients, respectively (95% CI: –16.7, 23.8; *P =* .841). No pyrazinamide C_max_ below the minimum reference range of 20 μg mL^–1^ was observed.

**Table 2:** Rifampicin, isoniazid and pyrazinamide area under the time‐concentration curve from 0 to 8 hours and maximum concentration: comparison of outpatient controls and hospitalized patients with human immunodeficiency virus‐associated tuberculosis

Drug	PK parameter	Outpatient controls	Hospitalized	*P*
		*n =* 48	*n =* 59	
Rifampicin	aAUC	43.8 [35.3, 53.8]	41.0 [28.3, 49.7]	.290
	bAUC	41.4 (1.5)	37.2 (1.8)	.291
	C_max_	8.3 [6.8, 9.5]	7.4 [6.1, 9.3]	.223
	Low C_max_	21 (43.8)	36 (62.1)	.079
Isoniazid	aAUC	12.4 [8.6, 18.9]	13.5 [8.9, 18.7]	.630
	bAUC	12.5 (1.6)	13.1 (1.7)	.632
	C_max_	3.5 [2.4, 4.5]	3.6 [2.6, 5.0]	.569
	Low C_max_	17 (35.4)	23 (39.0)	.841
Pyrazinamide	aAUC	292.2 [272.2, 319.3]	316.5 [255.4, 359.1]	.164
	bAUC	291.6 (1.8)	311.8 (1.3)	.165
	C_max_	46.8 (41.9, 51.1)	50.1 [44.1, 58.4]	.081
	Low C_max_	0 (0.0)	0 (0.0)	‐

PK: pharmacokinetic

a AUC: area under the time‐concentration curve from 0 to 8 hours in mg h L^–1^: median and interquartile range.

b AUC: area under the time‐concentration curve from 0 to 8 hours in mg h L^–1^: geometric mean and geometric standard deviation (approximate coefficient of variation).

C_max_: maximum concentration in μg mL^–1^: median and interquartile range.

Low C_max_: number and percentage of patients with maximum concentrations below minimum threshold of reference ranges: 8 μg mL^–1^ for rifampicin, 3 μg mL^–1^ for isoniazid and 20 μg mL^–1^ for pyrazinamide.^31^

*P* value represents the result of the nonparametric comparison (Wilcoxon rank sum test) for the numerical values or the Pearson's χ^2^ test for categorical variables, comparing outpatient controls to hospitalized patients with human immunodeficiency virus‐associated tuberculosis.

Comparing hospitalized patients who survived to those who died within 12 weeks, there were no significant differences in the median C_max_ for rifampicin (7.2 *vs* 7.5 μg mL^–1^, *P =* .655), isoniazid (3.9 *vs* 3.2 μg mL^–1^, *P =* .394) or pyrazinamide (48.0 *vs* 55.1 μg mL^–1^, *P =* .302; Table 3 and Figure 2). Comparing inpatients who survived to those who died, there was no difference in the proportion with low rifampicin C_max_ 29/47 (62%) *vs* 7/11 (64%; 95%CI: –35.5, 31.6; *P* > .999) and low isoniazid C_max_ 18/47 (38%) *vs* 5/11 (46%; 95%CI: –45.3, 31.0; *P =* .738; Figure 2 and Table 3).

**Table 3:** Rifampicin, isoniazid and pyrazinamide area under the time‐concentration curve from 0 to 8 hours and maximum concentration: comparison of hospitalized patients with human immunodeficiency virus‐associated tuberculosis who survived or died within 12 weeks

Drug	PK parameter	Hospitalized survivors	Hospitalized deaths	*P*
		*n =* 47	*n =* 11	
Rifampicin	aAUC	40.0 [27.8, 49.0]	43.2 (30.7, 49.4]	.684
	bAUC	35.5 (1.8)	41.5 (1.7)	.696
	C_max_	7.2 [6.1, 9.2]	7.5 [6.5, 9.1]	.655
	Low C_max_	29 (61.7)	7 (63.6)	>.999
Isoniazid	aAUC	13.4 [9.0, 18.4]	13.7 [7.1, 22.5]	.976
	bAUC	13.1 (1.7)	13.0 (2.0)	.984
	C_max_	3.9 [2.8, 5.0]	3.2 [2.5, 4.1]	.394
	Low C_max_	18 (38.1)	5 (45.5)	.738
Pyrazinamide	aAUC	310.9 [251.1, 354.2]	356.1 [293.0, 437.1]	.124
	bAUC	303.8 (1.3)	359.3 (1.4)	.128
	C_max_	48.0 [44.4, 57.6]	55.1 [45.0, 63.4]	.302
	Low C_max_	0 (0.0)	0 (0.0)	‐

Patients were followed up for 12 weeks to ascertain vital status. One patient was lost to follow up at 2 months and is not included in this table.

PK: pharmacokinetic

a AUC: area under the time‐concentration curve from 0 to 8 hours in mg h L^–1^: median and interquartile range.

b AUC: area under the time‐concentration curve from 0to 8 hours in mg h L^–1^: geometric mean and geometric standard deviation (approximate coefficient of variation).

C_max_: maximum concentration in μg mL^–1^: median and interquartile range.

Low C_max_: number and percentage of patients with maximum concentrations below minimum threshold of reference ranges: 8 μg mL^–1^ for rifampicin, 3 μg mL^–1^ for isoniazid and 20 μg mL^–1^ for pyrazinamide.^31^

*P* value represents the result of the nonparametric comparison (Wilcoxon rank sum test) for the numerical values or the Pearson's χ^2^ test for categorical variables, comparing hospitalized patients with human immunodeficiency virus‐associated tuberculosis who survived the 12‐weeks and those who died within 12 weeks.

### 3.3 AUC_0–8_

**Figure 3:**
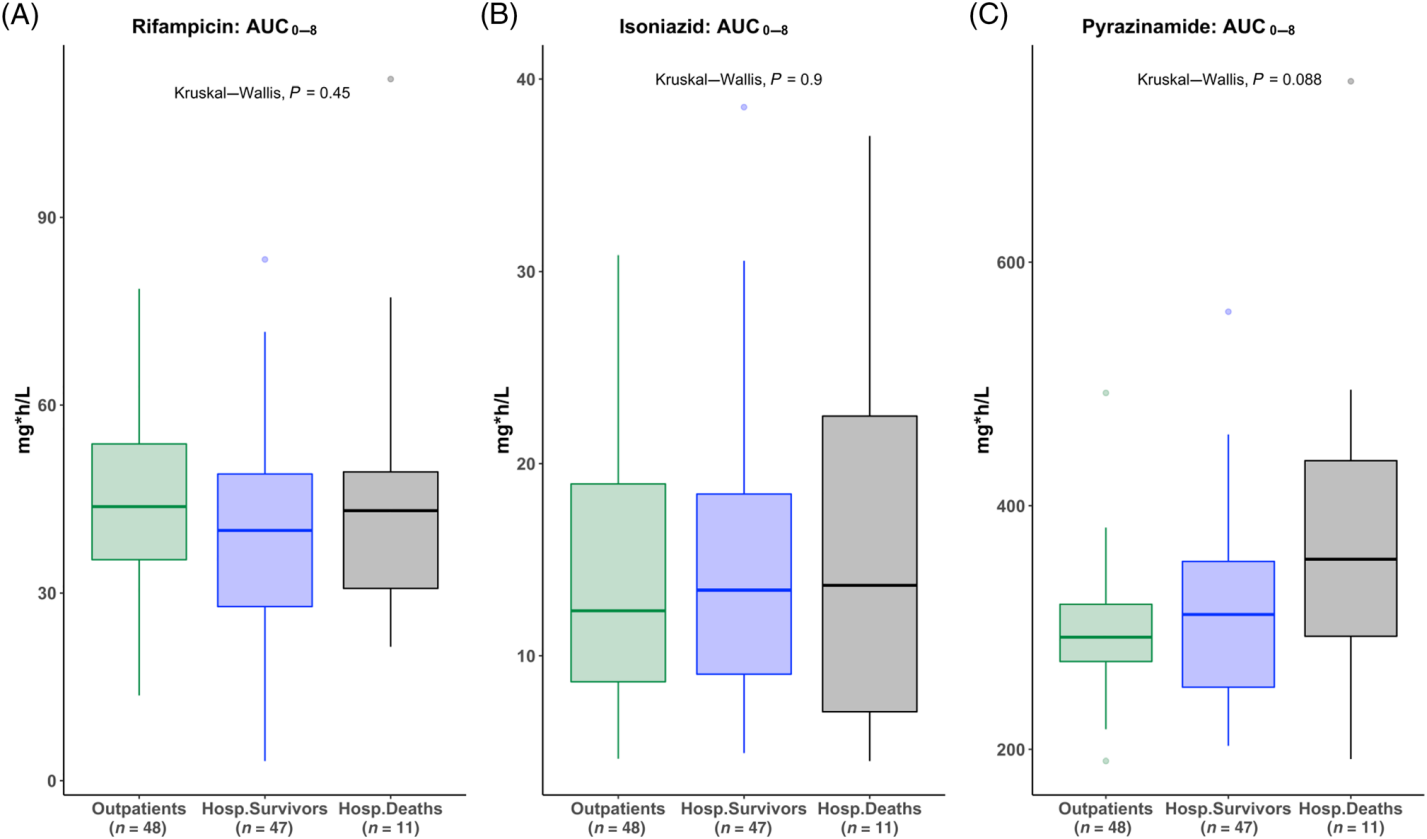
Rifampicin, isoniazid and pyrazinamide AUC_0‐8_ on the third day of antituberculosis therapy: boxplots of rifampicin, isoniazid and pyrazinamide AUC_0‐8_ presented in mg h L^–1^ for outpatients (green), hospitalized patients who survived 12 week follow up (blue) and hospitalized patients who died within 12 weeks (black). *P* value: Kruskal–Wallis test comparing AUC_0‐8_ values across 3 groups. AUC (0–8 h): area under the time‐concentration curve from 0 to 8 hours; Outpatients: ambulant tuberculosis patients attending outpatient clinic for treatment; Hosp.Survivors: hospitalized survivors; Hosp.Deaths: hospitalized patients who died within 12 weeks of enrolment

Comparing hospitalized patients to outpatients, the AUC_0–8_ for rifampicin (41.0 *vs* 43.8 mg h L^–1^, *P* = .290), isoniazid (13.5 *vs* 12.4 mg h L^–1^, *P =* .630) and pyrazinamide (316.5 *vs* 292.2 mg h L^–1^, *P =* .164) were not significantly different (Table 2 and Figure 3).

Hospitalized patients who survived and those who died within 12 weeks had similar AUC_0–8_ for rifampicin (40.0 *vs* 43.2 mg h L^–1^, *P =* .684), isoniazid (13.4 *vs* 13.7 mg h L^–1^, *P =* .976) and pyrazinamide (310.9 *vs* 356.1 mg h L^–1^, *P =* .128; Table 3 and Figure 3).

### 3.4 Patients presenting with an elevated lactate concentration

In hospitalized patients, venous lactate was performed at enrolment in 58/59 (98%) patients. One patient who survived did not have lactate performed.

Lactate was elevated (>2.2 mmol L^–1^) at presentation in 16/59 (27%). The median lactate for all inpatients was 1.6 mmol L^–1^, and the median was 1.45 mmol L^–1^ in patients who survived *vs* 2.4 mmol L^–1^ in patients who died, *P =* .078 (Table S3). The odds of survival decreased by 60% with doubling of the lactate concentration (odds ratio for survival per doubling of lactate: 0.41; 95% CI: 0.14, 1.08; *P =* .078). The proportion of patients presenting with an elevated lactate was 10/47 (21%) in survivors and 6/11 (55%; 95% CI: –4.0, 70.5; *P =* .079) in patients who died.

**Figure 4:**
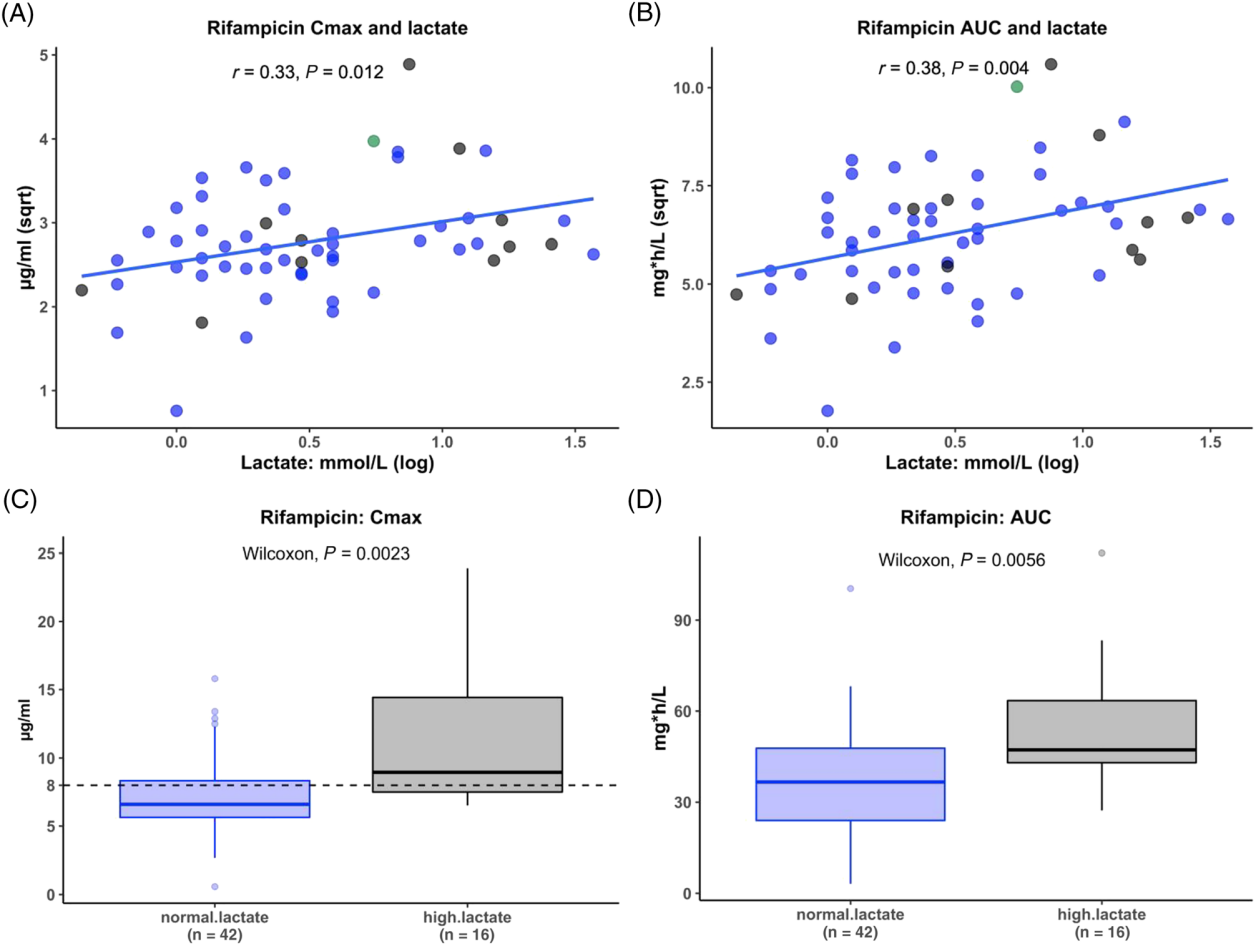
Rifampicin peak concentrations (C_max_) and area under the time‐concentration curve from 0 to 8 hours (AUC_0–8_) on the third day of antituberculosis therapy compared with lactate concentration at presentation. Rifampicin exposure was compared to lactate concentration, a marker of sepsis severity, at presentation. Rifampicin maximum concentration and area under the concentration time curve (AUC_0–8_) was first correlated with lactate concentration at presentation (panels A and B). The blue line indicates the linear regression line of best fit. The blue dots indicate patients who survived to 12 weeks, the black dots indicate patients who died within 12 weeks and the green dot indicates 1 patient who was lost to follow up at 8 weeks. Panels C and D are boxplots comparing rifampicin concentration in μg mL^–1^ between hospitalized patients who presented with a normal lactate concentration to those presenting with high lactate (>2.2 mmol L^–1^) concentrations. *r*: result of Spearman's rank correlation test in panel A and Pearson's correlation coefficient in panel B. C_max_: maximum (peak) concentration; Sqrt: square root transformed; log: log transformed; normal.lactate: patients presenting with normal lactate concentrations; high.lactate: patients presenting with high lactate (>2.2 mmol L^–1^)

Comparing clinical characteristics of patients presenting with an elevated lactate to patients with normal lactate, patients with elevated lactate had significantly higher random glucose (Table S4). Lactate concentration was positively correlated with random glucose and conjugated bilirubin concentrations (Table S4). Lactate concentration was positively correlated with rifampicin C_max_ and AUC_0–8_ with Spearman's rho of 0.329, *P =* .012 and 0.376, *P =* .004 respectively (Figure 4 and Table 4). Patients with an elevated lactate at presentation (>2.2 mmol L^–1^) had significantly higher rifampicin C_max_ (median = 9.0 *vs* 6.5 μg mL^–1^; *P =* .002) and AUC_0–8_ (median = 47.3 *vs* 36.7 mg h L^–1^; *P =* .006; Table 4) with a nonsignificant trend towards higher isoniazid and pyrazinamide C_max_ and AUC_0–8_ (Table 4). These findings are contrary to our hypothesis that patients presenting with elevated lactate would have lower exposure to TB drugs.

**Table 4:** Rifampicin, isoniazid and pyrazinamide area under the time‐concentration curve from 0 to 8 hours and maximum concentration: comparison of patients presenting with normal lactate and with high lactate concentrations

Drug	PK parameter	Normal lactate	High lactate	^1^ *P*	Correlation coefficient	^2^ *P*
		*n =* 42	*n =* 16			
Rifampicin	aAUC	36.7 [24.0, 47.8]	47.3 [43.0, 63.4]	**.006**	0.376	**.004**
	bAUC	32.9 (1.8)	50.6 (1.5)	**.003**	‐	‐
	C_max_	6.6 [5.6, 8.3]	9.0 [7.5, 14.4]	**.002**	0.329	**.012**
	Low C_max_	29 (70.7)	7 (43.8)	.073	‐	‐
Isoniazid	aAUC	13.2 [8.4, 17.8]	16.6 [9.7, 25.1]	.244	0.144	.281
	bAUC	12.6 (1.7)	15.2 (1.8)	.250	‐	‐
	C_max_	3.6 [2.6, 5.0]	4.1 [2.7, 5.2]	.424	0.096	.474
	Low C_max_	16 (38.1)	6 (37.5)	>.999	‐	‐
Pyrazinamide	aAUC	302.9 [234.7, 359.7]	333.1 [285.9, 369.2]	.117	0.162	.224
	bAUC	299.5 (1.3)	344.8 (1.3)	.120	‐	‐
	C_max_	47.4 [41.8, 57.6]	54.1 [47.2, 63.1]	.073	0.179	.180
	Low C_max_	0 (0.0)	0 (0.0)	‐	‐	‐

Lactate is used as a marker of sepsis severity and we divided patients into those presenting with high lactate (>2.2 mmol L^–1^, *n =* 16) and those presenting with normal lactate (*n =* 41) concentration. One patient who survived had no lactate performed and is not included in this table.

Pharmacokinetic parameters were compared between groups using a nonparametric comparison (Wilcoxon rank sum test) for the numerical values or the Pearson's χ^2^ test for categorical variables.

In addition, lactate was treated as a continuous variable and correlation tests (Spearman's rank correlation (distribution not normal) or Pearson's correlation test (normal distribution)) were used to correlate lactate concentrations with PK variables.

PK: pharmacokinetic

a AUC: area under the time‐concentration curve from 0 to 8 hours in mg h L^–1^: median and interquartile range.

b AUC: area under the time‐concentration curve from 0to 8 hours in mg h L^–1^: geometric mean and geometric standard deviation (approximate coefficient of variation).

C_max_: maximum concentration in μg mL^–1^: median and interquartile range.

Low C_max_: number and percentage of patients with maximum concentrations below minimum threshold of reference ranges: 8 μg mL^–1^ for rifampicin, 3 μg mL^–1^ for isoniazid and 20 μg mL^–1^ for pyrazinamide.^31^

Correlation coefficient: Spearman's ρ or Pearson's correlation coefficient.

#### 3.4.1 Associations of PK findings with selected clinical variables

Based on findings from previous studies and the physicochemical properties of the drugs we measured, we next performed an exploratory analysis to assess the relationship of selected clinical variables with our findings. In hospitalized patients we explored the correlations of PK variables with creatinine clearance and conjugated bilirubin concentrations. The only significant findings were a positive correlation of rifampicin AUC_0–8_ and C_max_ with creatinine clearance (Pearson's correlation coefficient [r] = 0.27, *P =* .040 and *r =* 0.29, *P =* .025 respectively) and a positive correlation of rifampicin AUC_0–8_ with conjugated bilirubin concentration (*r =* 0.38, *P =* .004). Rifampicin C_max_ and conjugated bilirubin showed a positive trend, *r =* 0.26, *P =* .055 (Figure S1).

Pyrazinamide exposure was not correlated with C‐reactive protein (AUC_0–8_: *r =* 0.02, *P =* .839, C_max_: *r =* 0.01, *P =* .885) or procalcitonin concentrations (AUC_0–8_: *r =* –0.01, *P =* .947, C_max_: *r =* 0.07, *P =* .585).

## 4 DISCUSSION

We measured concentrations of rifampicin, isoniazid and pyrazinamide on the third day of anti‐TB therapy in hospitalized adults with a new diagnosis of HIV‐TB and in outpatient controls. We found high 12‐week mortality of 19% for inpatients and no significant difference in C_max_ or AUC_0–8_ of rifampicin, isoniazid or pyrazinamide between hospitalized patients and outpatients, or between hospitalized patients who survived and those who died. Rifampicin and isoniazid peak concentrations were below reference ranges in 61% and 39% of inpatients and 44% and 35% of outpatients. All patients attained pyrazinamide concentrations within the reference range. We found significantly higher rifampicin C_max_ and AUC_0–8_ amongst patients presenting with elevated venous lactate, taken as a marker of sepsis severity.

We observed high 12‐week mortality despite treatment and patients died at a median of 40 days after the PK study. This time to death is longer than the median days to death in the main study, which was 12.5 days from enrolment.^34^ This PK study was performed within the routine clinical service. Critically ill patients requiring intensive care were transferred to a tertiary facility or died and stable patients were often discharged before the third day of anti‐TB therapy and could thus not be included in the PK study.

A large proportion of all patients had suboptimal rifampicin and isoniazid peak concentrations. Low concentrations of anti‐TB medications have been reported in other studies^29^, ^42^ and low exposure to pyrazinamide in particular have been associated with poor clinical outcomes. One study conducted intensive PK studies at 2 months on treatment and monitored 2‐year outcomes in South African pulmonary TB patients.^31^ They used classification and regression tree analysis, which identified pyrazinamide AUC_0–24_ < 363 mg h L^–1^ as the highest‐ranking factor associated with poor 2‐year outcomes (relapse, death or therapy failure). A predominantly HIV‐infected pulmonary TB cohort from Botswana had PK studies performed after at least 7 days on treatment and were followed for the duration of treatment. Lower peak concentrations of pyrazinamide (<35 μg mL^–1^) was the only PK variable associated with poor outcome and was associated with 3‐fold increased risk of poor outcome.^29^ In our cohort, pyrazinamide Cmax was <35 μg mL^–1^ in 6 patients (5 inpatients who survived and 1 outpatient) and there was a trend towards higher exposure in hospitalized patients. One potential mechanism for a trend towards higher pyrazinamide AUC_0–8_ in inpatients who died is impaired renal clearance due to acute kidney injury. Pyrazinamide and its main metabolite pyrazinoic acid are excreted in the urine^43^ and, although hospitalized patients and specifically inpatients who died had higher creatinine, we observed no significant correlation between pyrazinamide exposure and creatinine clearance. Pyrazinamide clearance was shown to be inversely correlated to chronic cellular immune activation in HIV‐TB patients in Botswana.^44^ We did not measure human leucocyte antigen‐DR expression on CD8 T cells in our study and, even though hospitalized patients and specifically patients who died had higher C‐reactive protein and procalcitonin, there was no significant correlation between pyrazinamide exposure and either of these markers.

In a previous study, optimal early bactericidal activity was associated with an isoniazid C_max_ and AUC_0–∞_ of >2.19 and 10.52 mg h L^–1^, respectively. In our study 10/59 (17%) and 23/59 (40%) of hospitalized patients had isoniazid C_max_ and AUC_0–8_ below these values respectively. Optimal early bactericidal effect may be important for survival in this patient group.

In hospitalized patients, we found a median rifampicin AUC_0–8_ of 41.0 mg h L^–1^, which is higher than the predicted AUC_0–24_ (30.7 mg h L^–1^) previously reported in South African pulmonary TB patients and HIV‐infected pulmonary TB patients in Botswana at steady state (36.3 and 34.4 mg h L^–1^).^42^, ^45^, ^46^ Higher AUC_0–8_ values in our study are expected because auto‐induction with the resulting drop in AUC value takes 4 weeks^47^, ^48^ and would not have been complete at this early therapeutic time point (3 days).

We found significantly higher C_max_ and AUC_0–8_ for rifampicin in inpatients presenting with elevated lactate, which is contrary to our hypothesis, and a positive correlation of rifampicin C_max_ and AUC_0–8_ with conjugated bilirubin. Lactate is used as a marker of sepsis severity and probably reflects increased aerobic glycolysis on cellular level due to adrenergic stimulation^49^ and metabolic switching of activated innate immune cells to aerobic glycolysis in critically ill patients.^50^, ^51^ The largest proportion of tissue resident macrophages (Kupffer cells) are present in the liver and these cells play a critical role in the innate immune response to pathogens, which involves activation and metabolic switch to a proinflammatory (M1‐macrophage) phenotype and have important antimicrobial activity.^52^ Rifampicin and its active metabolite desacetylrifampicin are lipid soluble, have enterohepatic circulation, competes with bilirubin for biliary excretion and are excreted mainly in bile and but also in urine.^32^ High pretreatment bilirubin levels in patients with advanced liver cirrhosis are associated with higher rifampicin exposure.^53^ It is possible that the cellular metabolic changes which underly higher lactate concentrations could play a role in the higher rifampicin exposure we observed in these patients. Neither of the hydrophylic drugs (isoniazid or pyrazinamide) were correlated with creatinine clearance, but rifampicin exposure was positively correlated with creatinine clearance. The mechanism for this is unclear.

One third of hospitalized patients in this cohort had *M. tuberculosis* blood stream infection; however, inpatients achieved concentrations and exposures similar to ambulant outpatients. The high mortality amongst hospitalized patients and the high proportion with maximum concentrations below minimum thresholds of reference ranges suggest that these concentrations and exposures may not be adequate in critically ill patients.

The parent study demonstrated an association between mortality and a higher number of mycobacterial dissemination markers being positive as well as an immune profile dominated by innate mediators.^34^ These findings together with our findings of sub‐optimal rifampicin and isoniazid concentrations in hospitalized HIV‐TB patients provide directions to consider for improving treatment strategies. One objective of treatment optimization studies should be to evaluate if more rapid reduction of disseminated mycobacterial infection load can be achieved and whether this improves survival. Strategies to accomplish this could include higher dose rifampicin, higher dose isoniazid or the addition of another rapidly bactericidal drug such as a fluoroquinolone. The safety and efficacy of these strategies would need to be tested in clinical trials in this patient population. Other strategies to evaluate could include those that modulate the immune response associated with mortality. Treatment optimization research in hospitalized HIV‐TB patients should consider and could draw on the experience of treatment optimization research in the field of tuberculous meningitis, in particular, findings regarding the efficacy and safety of higher dose rifampicin and isoniazid.^54^

Strengths of this study are that we performed intensive PK sampling in acutely ill hospitalized patients in a routine care setting at an early therapeutic time point and in a control group with pulmonary TB from the same geographical area at the same therapeutic time point. These results could help to inform future treatment optimization strategies in hospitalized HIV‐TB patients. The study has several limitations. Firstly, performing the PK studies on the third day of anti‐TB therapy probably introduced survival bias because some critically ill patients were transferred or died before the third day of anti‐TB therapy. Half of the hospitalized patients received an FDC from a different manufacturer and this may have introduced variation in the drug concentrations. We did not calculate AUC_0–24_ and reported only AUC_0–8_. We did not perform genotyping to assess patients' isoniazid acetylator status. Potential unmeasured differences in distribution of acetylator status across the comparator groups may have biased our analysis of the PK of isoniazid. The associations of PK variables with selected clinical variables could be due to other underlying mechanisms than the potential mechanisms we explored.

In conclusion, rifampicin and isoniazid peak concentrations were below reference ranges in 62% and 39% of hospitalized patients with HIV‐TB, respectively. Isoniazid peak concentration and exposure were below the levels associated with optimal early bactericidal activity in 17% and 40% of inpatients, respectively. Inadequate exposure to key anti‐TB drugs during initial therapy may contribute to the high mortality observed in acutely ill patients hospitalized with HIV‐TB. While upstream public health interventions are needed to prevent diagnostic and treatment delays, TB treatment strategies in patients hospitalized with HIV‐TB need to be optimized to improve survival. Novel therapeutic strategies should be evaluated for safety and efficacy in this patient population.

## COMPETING INTERESTS

There are no competing interests to declare.

## CONTRIBUTORS

Gr.M. conceptualized the study with input from Ga.M., R.J.W., H.M. and P.D. Gr.M. funded the study. C.S. and A.W. recruited patients and performed PK sampling with assistance from S.J. and D.B. Gr.M. and R.B. provided clinical oversight of recruitment. R.J.W. provided laboratory facilities for storages of samples and MS provided laboratory oversight. L.W. provided oversight of measurement of drug concentrations. C.S. curated drug concentration results and analysed data with assistance of D.B. and M.C. C.S. wrote the manuscript and all coauthors reviewed and contributed to the manuscript.

## Supporting information


**FIGURE S1** Rifampicin maximum concentration and area under the concentration curve: correlation with creatinine clearance and conjugated bilirubin.

**TABLE S1** Missing pharmacokinetic study time points: samples not collected
**TABLE S2** Missing pharmacokinetic study time points: value below the lower limit of quantification
**TABLE S3** Baseline characteristics of hospitalized patients with human immunodeficiency virus‐associated tuberculosis who had intensive pharmacokinetic studies performed on the third day of antituberculosis therapy: comparison between patients who died within 12 weeks and those who survived

## Data Availability

The data that support the findings of this study are openly available in ZivaHub Open Data UCT by FigShare] at https://doi.org/10.25375/uct.9541991.v1, title: Khayelitsha Hospital TB study PK variables: Non compartmental analysis.
