# Supplementary figures and images for: Early antituberculosis drug exposure in hospitalized patients with human immunodeficiency virus‐associated tuberculosis
Source: Br J Clin Pharmacol. 2020 Feb 17;86(5):966–78. doi: 10.1111/bcp.14207 (PMC7163385; doi:10.1111/bcp.14207)

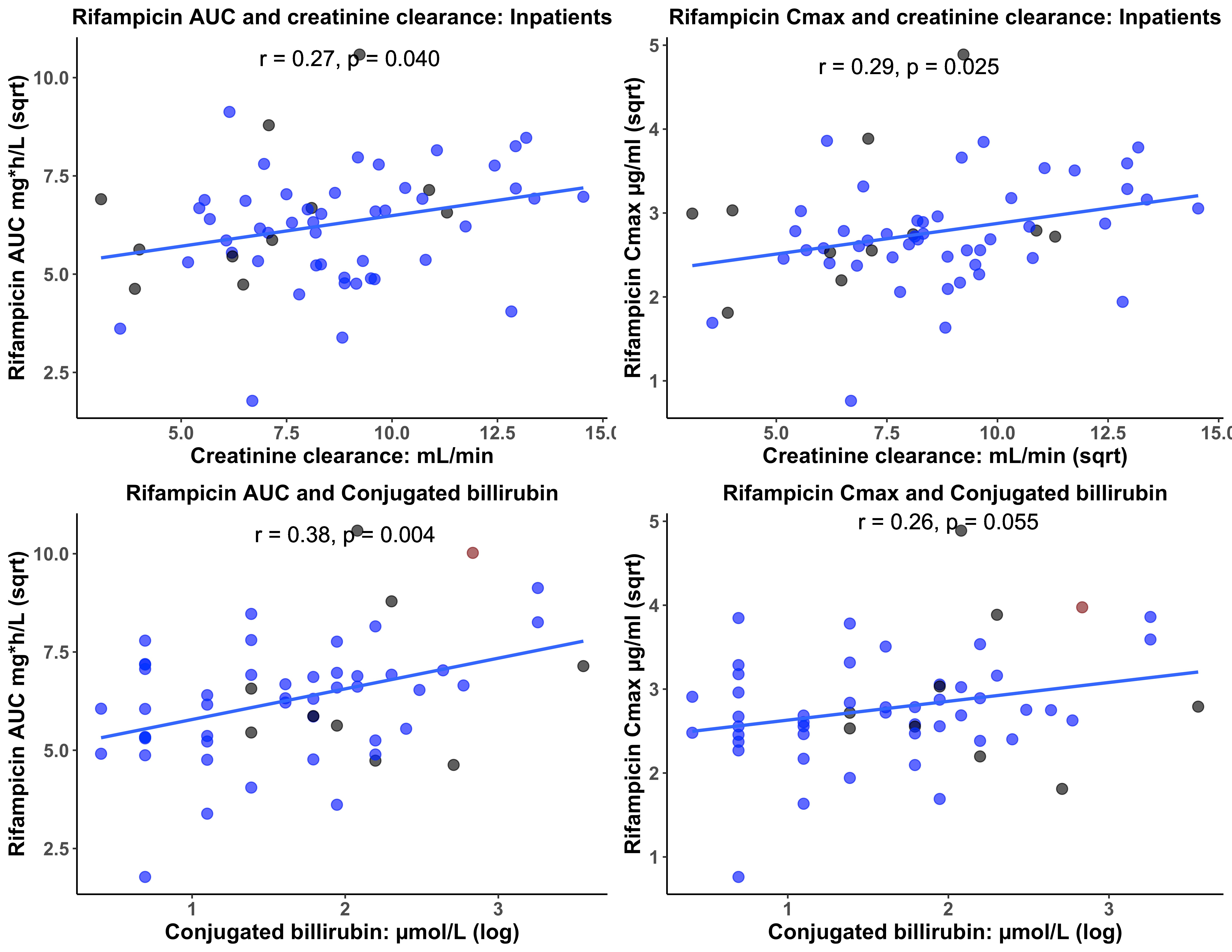

Supplement: Supplementary file 1 — FIGURE S1 Rifampicin maximum concentration and area under the concentration curve: correlation with creatinine clearance and conjugated bilirubin. [file BCP-86-966-s001.docx]
