# Supplementary material for: Early antituberculosis drug exposure in hospitalized patients with human immunodeficiency virus‐associated tuberculosis
Source: Br J Clin Pharmacol. 2020 Feb 17;86(5):966–78. doi: 10.1111/bcp.14207 (PMC7163385; doi:10.1111/bcp.14207)
Supplement: Supplementary file 2 — TABLE S1 Missing pharmacokinetic study time points: samples not collected TABLE S2 Missing pharmacokinetic study time points: value below the lower limit of quantification TABLE S3 Baseline characteristics of hospitalized patients with human immunodeficiency virus‐associated tuberculosis who had intensive pharmacokinetic studies performed on the third day of antituberculosis therapy: comparison between patients who died within 12 weeks and those who survived [file BCP-86-966-s002.docx]

**Supplementary Table 1: Missing pharmacokinetic study time points: Samples not collected:**

| PK time point | Medication | Samples not taken  n/N  N=107 | Value imputed  n/missing | Outpatient controls  n=48 | Inpatient Survivors  n=47 | Inpatient Deaths  n=11 |
| --- | --- | --- | --- | --- | --- | --- |
| 0 hour | Rifampicin | 0 (0%) | - | - | - | - |
|  | Isoniazid | 0 (0%) | - | - | - | - |
|  | Pyrazinamide | 0 (0%) | - | - | - | - |
| 1 hour | Rifampicin | 0 (0%) | - | - | - | - |
|  | Isoniazid | 0 (0%) | - | - | - | - |
|  | Pyrazinamide | 0 (0%) | - | - | - | - |
| 2.5 hour | Rifampicin | 2 (2%) | 0 (0%) | 1 (2%) | 1 (2%) | 0 (0%) |
|  | Isoniazid | 2 (2%) | 0 (0%) | 1 (2%) | 1 (2%) | 0 (0%) |
|  | Pyrazinamide | 2 (2%) | 0 (0%) | 1 (2%) | 1 (2%) | 0 (0%) |
| 4 hour | Rifampicin | 2 (2%) | 0 (0%) | 2 (4%) | 0 (0%) | 0 (0%) |
|  | Isoniazid | 2 (2%) | 0 (0%) | 2 (4%) | 0 (0%) | 0 (0%) |
|  | Pyrazinamide | 2 (2%) | 0 (0%) | 2 (4%) | 0 (0%) | 0 (0%) |
| 6 hour | Rifampicin | 0 (0%) | - | - | - | - |
|  | Isoniazid | 1 (1%) | 0 (0%) | 0 (0%) | 1 (2%) | 0 (0%) |
|  | Pyrazinamide | 0 (0%) | - | - | - | - |
| 8 hour | Rifampicin | 11 (10%) | 10 (91%) | 6 (13%) | 2 (4%) | 2 (18%) |
|  | Isoniazid | 11 (10%) | 11 (100%) | 7 (15%) | 2 (4%) | 2 (18%) |
|  | Pyrazinamide | 11 (10%) | 11 (100% | 7 (15%) | 2 (4%) | 2 (18%) |

PK time point: Time at which the samples were collected in relation to administration of the study medication.

Medication: Indicates antituberculosis drug at each time point.

Samples not taken: Indicate the total number of samples at each time which were not collected and indicates the percentage value of the missing samples to expected samples at each time point.

N= Number of expected samples at each time point which is 107.

Value imputed: Indicates number of values which were imputed using the log concentration curve for each patient and gives the percentage of imputed values to missing values at each time point.

Outpatient controls, Inpatient Survivors and Inpatient Deaths indicates the spread of missing samples by group and percentage values of missing samples to the total expected samples in each group.

**Supplementary Table 2: Missing pharmacokinetic study time points: Value below the lower limit of quantification:**

| PK time point | Medication | Below LLQ  n/N  N= 107 | Outpatient Controls  n=48 | Inpatient Survivors  n=47 | Inpatient Deaths  n=11 |
| --- | --- | --- | --- | --- | --- |
| 0 hour | Rifampicin | 30 (28%) | 13 (27%) | 16 (34%) | 1 (9%) |
|  | Isoniazid | 76 (71%) | 30 (62%) | 36 (77%) | 9 (82%) |
|  | Pyrazinamide | 1 (1%) | 0 (0%) | 1 (2%) | 0 (0%) |
| 1 hour | Rifampicin | 1 (1%) | 0 (0%) | 1 (2%) | 0 (0%) |
|  | Isoniazid | 2 (2%) | 1 (2%) | 1 (2%) | 0 (0%) |
|  | Pyrazinamide | 0 (0%) | - | - | - |
| 2.5 hour | Rifampicin | 0 (0%) | - | - | - |
|  | Isoniazid | 0 (0%) | - | - | - |
|  | Pyrazinamide | 0 (0%) | - | - | - |
| 4 hour | Rifampicin | 0 (0%) | - | - | - |
|  | Isoniazid | 0 (0%) | - | - | - |
|  | Pyrazinamide | 0 (0%) | - | - | - |
| 6 hour | Rifampicin | 0 (0%) | - | - | - |
|  | Isoniazid | 1 (1%) | 0 (0%) | 1 (2%) | 0 (0%) |
|  | Pyrazinamide | 0 (0%) | - | - | - |
| 8 hour | Rifampicin | 0 (0%) | - | - | - |
|  | Isoniazid | 8 (8%) | 2 (4%) | 6 (13%) | 0 (0%) |
|  | Pyrazinamide | 0 (0%) | - | - | - |

PK time point: Time at which the samples were collected in relation to administration of the study medication.

Medication: Indicates antituberculosis drug at each time point.

Below LLQ: Indicates the total number of samples at each time point which were below the lower limit of quantification. The percentage value is the number of samples below the LLQ to total expected samples at each time point.

N= Number of expected samples at each time point which is 107.

Outpatient controls, Inpatient Survivors and Inpatient Deaths indicates the spread of samples below the LLQ by group. The percentage indicates number of samples below LLQ to the total expected samples in each group.

LLQ: Lower limit of quantification,

LLQ for rifampicin = 0.117 μg/ml

LLQ for isoniazid = 0.105 μg/ml

LLQ for pyrazinamide = 0.203 μg/ml

Half of the value of the LLQ was imputed for all concentrations below LLQ for the analysis.

**Supplementary Table 3: Baseline characteristics of hospitalized patients with HIV-associated tuberculosis who had intensive pharmacokinetic studies performed on the third day of antituberculosis therapy: Comparison between patients who died within twelve weeks and those who survived:**

|  | **Hospitalized Survivors**  **n=47** | **Hospitalized Deaths**  **n=11** | **p** |
| --- | --- | --- | --- |
| First episode of TB | 26 (56.5) | 8 (72.7) | 0.592 |
| Sex, Male | 23 (48.9) | 5 (45.5) | 1.000 |
| Age, years | 38 [32, 40] | 35 [31, 50] | 0.960 |
| HIV viral load, log copies ml^-1^ | 4.9 [2.9, 5.6] | 5.6 [5.3, 6.0] | 0.051 |
| CD4 count, cells μL^-1^ | 77 [18, 132] | 32 [13, 94] | 0.275 |
| Current antiretroviral therapy | 19 (41.3) | 1 (9.1) | **0.049** |
| MTB on TB blood culture | 13 (28.9) | 3 (27.3) | 1.000 |
| Glasgow coma score <15 at presentation | 7 (14.9) | 5 (45.5) | **0.039** |
| ^a^Height, meters | 1.64 [1.59, 1.70] | 1.64 [1.59, 1.71] | 0.736 |
| Weight, kilograms | 54.0 [48.0, 60.5] | 55.0 [47.5, 60.0] | 0.843 |
| ^b^Body mass index | 19.3 [17.4, 22.3] | 21.7 [18.6, 23.1] | 0.300 |
| Body mass index <18.5 kg per m^2^ | 19 (44.2) | 2 (25.0) | 0.340 |
| Body mass index 18.5 – 24.9 kg per m^2^ | 21 (48.8) | 5 (62.5) |  |
| Body mass index >25 kg per m^2^ | 3 (6.4) | 1 (.1) |  |
| Random glucose, mmol L^-1^ | 5.1 [4.6, 5.5] | 6.4 [5.3, 7.3] | **0.006** |
| Lactate, mmol L^-1^ | 1.45 [1.10, 1.80] | 2.40 [1.50, 3.35] | 0.078 |
| C-reactive protein, mg L^-1^ | 175.0 [101.1, 246.0] | 231.3 [182.5, 316.8] | 0.076 |
| Procalcitonin, μg mL^-1^ | 2.3 [0.5, 18.2] | 10.1 [4.0, 15.5] | 0.129 |
| Aspartate amino transferase, U L^-1^ | 47.0 [33.5, 83.5] | 64.5 [36.3, 81.0] | 0.632 |
| Alanine amino transferase, UL^-1^ | 30.0 [20.0, 49.5] | 22.0 [15.0, 30.5] | 0.100 |
| Gamma-glutamyl transferase, U L^-1^ | 77.0 [50.5, 126.5] | 90.0 [40.0, 393.0] | 0.388 |
| Alkaline phosphatase, U L^-1^ | 99.0 [73.0, 148.5] | 129.0 [113.5, 263.5] | 0.015 |
| Total bilirubin, μmol L^-1^ | 9.0 [5.0, 14.0] | 12.0 [9.5, 15.0] | 0.136 |
| Conjugated bilirubin, μmol L^-1^ | 5.0 [2.0, 8.0] | 8.0 [6.0, 10.0] | **0.033** |
| Total protein, g L^-1^ | 80.0 [72.0, 86.0] | 80.0 [67.0, 81.0] | 0.262 |
| Albumin, g L^-1^ | 26.0 [22.0, 30.5] | 22.0 [16.5, 25.0] | **0.031** |
| Creatinine, μmol L^-1^ | 90.0 [63.0, 131.5] | 136.00 [78.0, 315.5] | 0.102 |
| Creatinine clearance, mL minute^-1^ | 74.8 [47.9, 101.6] | 50.1 [27.4, 75.4] | 0.079 |
| Haemoglobin, g dL^-1^ | 9.0 [7.5, 10.1] | 7.7 [6.0, 8.8] | 0.022 |
| White cell count, x10^9^ L^-1^ | 7.1 [5.5, 9.5] | 7.7 [4.1, 12.4] | 0.913 |
| Platelets, x10^9^ L^-1^ | 299.0 [213.5, 360.0] | 204.0 [175.5, 318.5] | 0.262 |
| Absolute neutrophil count, x10^9^ L^-1^ | 5.3 [3.6, 8.0] | 7.0 [3.6, 10.3] | 0.781 |
| Absolute lymphocyte count, x10^9^ L^-1^ | 0.71 [0.4, 1.2] | 0.4 [0.3, 0.7] | 0.100 |
| Absolute monocyte count, x10^9^ L^-1^ | 0.4 [0.2, 0.7] | 0.2 [0.1, 0.4] | 0.065 |
| Rifampicin dose, mg kg^-1^ | 10.0 [9.3 - 11.1] | 10.0 [8.9 - 10.7] | 0.766 |
| Isoniazid dose, mg kg^-1^ | 5.0 [4.7 - 5.6] | 5.0 [4.5 - 5.4] | 0.662 |
| Pyrazinamide dose, mg kg^-1^ | 26.7 [24.9 - 29.6] | 26.2 [23.7 - 28.6] | 0.713 |

Supplementary Table 3: Baseline characteristics of hospitalized patients comparing patients who survived 12 weeks of follow up and patients who died within 12 weeks.

One patient was lost to follow up at 2 months and is not included in this table.

Continuous variables are presented as median with interquartile range and categorical variables as number with percentage.

p-value represents result of the non-parametric test comparison (Wilcoxon rank sum test for continuous variables and Fisher’s exact or Pearson’s Chi squared test for categorical variables)

TB: Tuberculosis; HIV: Human immunodeficiency virus; CD4: Cluster of differentiation 4; MTB: *Mycobacterium tuberculosis*

^a^Height was missing in 8 patients: 4 survivors, 3 patients who died, 1 lost to follow

^b^BMI was not calculated for patients with missing height

**Supplementary Table 4: Baseline characteristics of hospitalized patients with HIV-associated tuberculosis who had intensive pharmacokinetic studies performed on the third day of antituberculosis therapy: Comparison between patients with high lactate (> 2.2 mmol L^-1^) and those with normal lactate at presentation.**

| **Clinical characteristic** | **High lactate**  **n=16** | **Normal lactate**  **n=42** | **^1^p** | **Correlation**  **coefficient** | **^2^p** |
| --- | --- | --- | --- | --- | --- |
| First episode of TB | 13 (81.2) | 21 (50.0) | 0.084 | - | 0.176 |
| Sex, Male | 7 (43.8) | 21 (50.0) | 0.772 | - | 0.870 |
| Age, years | 38 [31, 45] | 37 [32, 40] | 0.808 | 0.027 | 0.842 |
| HIV viral load, log copies ml^-1^ | 5.3 [4.6, 5.8] | 4.9 [3.3, 5.6] | 0.350 | 0.141 | 0.301 |
| CD4 count, cells μL^-1^ | 64 [19, 115] | 45 [14, 142] | 0.801 | -0.009 | 0.944 |
| Current antiretroviral therapy | 4 (25.0) | 15 (36.6) | 0.171 | - | 0.166 |
| MTB on TB blood culture | 4 (26.7) | 11 (26.8) | 1.000 | - | 0.677 |
| Glasgow coma score <15 at presentation | 6 (37.5) | 6 (14.3) | 0.072 | - | 0.346 |
| ^a^Height, meters | 1.64 [1.59, 1.69] | 1.64 [1.59, 1.70] | 0.931 | -0.074 | 0.612 |
| Weight, kilograms | 57.1 [44.0, 65.5] | 54.0 [48.0, 59.0] | 0.450 | 0.074 | 0.584 |
| ^b^Body mass index | 20.5 [16.1, 23.6] | 19.3 [17.8, 21.8] | 0.666 | 0.056 | 0.700 |
| Body mass index <18.5 kg per m^2^ | 6 (42.9) | 15 (41.7) | 0.083 | - | 0.325 |
| Body mass index 18.5 – 24.9 kg per m^2^ | 5 (35.7) | 20 (55.6) |  |  |  |
| Body mass index >25 kg per m^2^ | 3 (18.8) | 1 (2.4) |  |  |  |
| Random glucose, mmol L^-1^ | 6.1 [4.7, 7.3] | 5.1 [4.7, 5.4] | **0.038** | **0.367** | **0.005** |
| C-reactive protein, mg L^-1^ | 183.5 [104.8, 252.5] | 197.0 [109.3, 296.8] | 0.596 | -0.052 | 0.696 |
| Procalcitonin, μg mL^-1^ | 3.4 [0.9, 10.9] | 4.0 [0.6, 22.5] | 0.657 | 0.016 | 0.903 |
| Aspartate amino transferase, U L^-1^ | 78.0 [35.0, 89.0] | 43.5 [34.0, 70.0] | 0.268 | 0.187 | 0.189 |
| Alanine amino transferase, U L^-1^ | 27.5 [17.3, 36.3] | 26.5 [18.5, 49.0] | 0.632 | -0.066 | 0.624 |
| Gamma-glutamyl transferase, U L^-1^ | 83.5 [37.8, 170.5] | 75.0 [52.5, 132.8] | 0.876 | -0.054 | 0.690 |
| Alkaline phosphatase, U L^-1^ | 125.5 [76.5, 191.8] | 99.0 [74.0, 147.0] | 0.174 | 0.133 | 0.324 |
| Total bilirubin, μmol L^-1^ | 10.5 [6.8, 14.0] | 10.0 [6.0, 15.0] | 0.944 | 0.142 | 0.288 |
| Conjugated bilirubin, μmol L^-1^ | 7.0 [4.0, 9.0] | 5.0 [3.0, 9.0] | 0.304 | **0.267** | **0.051** |
| Total protein, g L^-1^ | 80.0 [70.0, 83.3] | 79.0 [71.0, 86.0] | 0.882 | -0.039 | 0.769 |
| Albumin, g L^-1^ | 23.5 [19.8, 29.8] | 25.5 [22.0, 29.8] | 0.519 | -0.102 | 0.445 |
| Creatinine, μmol L^-1^ | 80.5 [59.0, 189.5] | 69.1 [44.7, 96.8] | 0.958 | -0.148 | 0.269 |
| Creatinine clearance, mL minute^-1^ | 66.4 [48.2, 87.4] | 69.1 [44.7, 96.8] | 0.972 | 0.073 | 0.588 |
| Haemoglobin, g dL^-1^ | 7.90 [7.07, 8.93] | 9.1 [7.5, 10.3] | 0.117 | -0.136 | 0.307 |
| White cell count, x10^9^ L^-1^ | 7.45 [3.58, 12.39] | 7.2 [5.5, 9.4] | 0.767 | -0.019 | 0.889 |
| Platelets, x10^9^ L^-1^ | 253 [159, 306] | 303 [219, 361] | 0.167 | -0.129 | 0.335 |
| Absolute neutrophil count, x10^9^ L^-1^ | 7.60 [3.18, 10.73] | 5.4 [4.1, 7.6] | 0.808 | 0.123 | 0.359 |
| Absolute lymphocyte count, x10^9^ L^-1^ | 0.50 [0.25, 0.77] | 0.70 [0.37, 1.07] | 0.220 | -0.170 | 0.201 |
| Absolute monocyte count, x10^9^ L^-1^ | 0.23 [0.15, 0.51] | 0.38 [0.20, 0.64] | 0.189 | -0.177 | 0.183 |
| Died within 12 weeks | 6 (37.5) | 5 (11.9) | 0.079 | - | - |

Supplementary Table 4:

One patient who survived had no lactate performed and is not included in this table.

Median and interquartile range are presented for continuous variables and number with percentage for categorical variables.

TB: Tuberculosis; HIV: Human immunodeficiency virus; CD4: Cluster of differentiation 4; MTB: *Mycobacterium tuberculosis*

Patients were divided into those presenting with raised (n=16) or normal lactate (n=41) and baseline characteristics were compared using non-parametric (Wilcoxon rank sum test for continuous variables and Fisher’s exact or Pearson’s Chi squared test for categorical variables) tests.

^1^p represents the result of the comparison of the baseline characteristic values between patients presenting with high or normal lactate.

In addition, lactate was treated as a continuous variable and correlation tests (Spearman’s rank correlation or Pearson’s correlation test) were used to correlate lactate concentrations with continuous baseline clinical variables. Non-parametric tests (Wilcoxon rank sum or Kruskal Wallis test) were used to compare lactate concentrations across different levels of categorical variables.

Correlation coefficient: Spearman’s rho (if normal distribution not achieved by log or square root transformation) or Pearson’s correlation coefficient (if both variables were normally distributed).

^2^p represents the result of the correlation test or the non-parametric test comparing lactate concentration to the baseline characteristic.

^a^Height was missing in 2 patients with high lactate and 6 patients with normal lactate.

^b^BMI was not calculated for patients with missing height.

**Supplementary Table 5: Rifampicin, isoniazid and pyrazinamide area under the concentration curve (0-8 hours) and maximum concentration: Comparison of outpatient controls tuberculosis stratified by HIV-status:**

| **Drug** | **PK parameter** | **Outpatient Control**  **HIV negative**  **n=19** | **Outpatient Control**  **HIV positive**  **n=29** | **p** |
| --- | --- | --- | --- | --- |
| **Rifampicin** | **AUC** | **40.6 [33.9, 50.1]** | **47.1 [35.8, 56.7]** | **0.202** |
|  | **C_max_** | **8.2 [6.5, 8.9]** | **8.8 [6.9, 10.5]** | **0.255** |
|  | **Low C_max_** | **9 (47.4)** | **12 (41.4)** | **0.770** |
| **Isoniazid** | **AUC** | **12.3 [9.0, 20.0]** | **12.8 [8.1, 17.0]** | **0.728** |
|  | **C_max_** | **3.7 [2.4, 4.6]** | **3.4 [2.5, 4.5]** | **0.628** |
|  | **Low C_max_** | **6 (31.6)** | **11 (37.9)** | **0.762** |
| **Pyrazinamide** | **AUC** | **290.8 [272.5, 305.8]** | **292.4 [272.3, 327.7]** | **0.487** |
|  | **C_max_** | **45.7 [42.6, 52.1]** | **47.1 [41.9, 50.8]** | **0.874** |
|  | **Low C_max_** | **0 (0.0)** | **0 (0.0)** | **-** |

PK: Pharmacokinetic; n= number

AUC: Area under the concentration curve: 0 – 8 hours in mg·h L^-1^: Median and interquartile range are presented.

C_max_: Maximum concentration in μg ml^-1^: Median and interquartile range are presented.

Low C_max_: Number and percentage of patients with maximum concentrations below minimum threshold of reference ranges: 8 μg mL^-1^ for rifampicin, 3 μg mL^-1^ for isoniazid and 20 μg mL^-1^ for pyrazinamide (reference: Alsultan A, Peloquin CA. Therapeutic drug monitoring in the treatment of tuberculosis: an update. Drugs. 2014;74(8):839-54).

P value represents the result of the non-parametric comparison (Wilcoxon rank sum test) for the numerical values or the Pearson’s Chi squared test for categorical variables, comparing outpatient controls with tuberculosis who were HIV negative to those who were HIV positive.
